# Chemical Vapor Deposition of N-Doped Graphene through Pre-Implantation of Nitrogen Ions for Long-Term Protection of Copper

**DOI:** 10.3390/ma14133751

**Published:** 2021-07-05

**Authors:** Luoqiao Han, Lei Dong, Haiyan Chen, Shuai Yang, Aiheng Yuan, Ran Guan, Hong Yan, Jing Wu, Bo Zhang, Dejun Li, Birong Luo

**Affiliations:** 1 College of Physics and Materials Science, Tianjin Normal University, Tianjin 300387, China; luoqiaohan@hotmail.com (L.H.); dlei0008@tjnu.edu.cn (L.D.); shuaiyang999@gmail.com (S.Y.); yuanyah@hotmail.com (A.Y.); guanran254@gmail.com (R.G.); yanhong04010603@gmail.com (H.Y.); wujingwu97@gmail.com (J.W.); zhangbo2014@tjnu.edu.cn (B.Z.); dejunli@tjnu.edu.cn (D.L.); 2 Fujian College of Water Conservancy and Electric Power, Yongan 366000, China

**Keywords:** chemical vapor deposition, N-doped graphene, coating, implantation, oxidation, corrosion, long-term protection

## Abstract

Nitrogen-doped graphene (NG) was synthesized through the chemical vapor deposition (CVD) of graphene on Cu substrates, which were pre-implanted with N ions by the ion implantation method. The pre-implanted N ions in the Cu substrate could dope graphene by the substitution of C atoms during the CVD growth of graphene, forming NG. Based on this, NG’s long-term protection properties for Cu were evaluated by ambient exposure for a corrosion test. The results showed that NG can obviously reduce the natural oxidation of Cu in the long-term exposure compared with the case of pristine graphene (PG) coated on Cu. Moreover, with the increase in pre-implanted N dose, the formed NG’s long-term protection for Cu improved. This indicates that the modification of graphene by N doping is an effective way to improve the corrosion resistance of the PG coating owing to the reduction in its conductivity, which would inhibit galvanic corrosion by cutting off electron transport across the interface in their long-term protection. These findings provide insight into corrosion mechanisms of the graphene coating and correlate with its conductive nature based on heteroatoms doping, which is a potential route for improving the corrosion resistance of graphene as an effective barrier coating for metals.

## 1. Introduction

Graphene is a typical two-dimensional (2D) material with a tight hexagonal structure and many kinds of outstanding properties such as high strength, excellent thermal conductivity, and superior impermeability to gases [1,2,3,4]. These properties make graphene an excellent corrosion barrier for metals, because it acts as a good physical barrier, being chemically inert, preventing metals from coming into contact with O_2_ and H_2_O in the surroundings [5,6,7,8,9]. However, it has been revealed that the graphene coating provides corrosion protection for the metal only for a short period of time. For a long period of time, the galvanic corrosion between the graphene and the metal will accelerate the corrosion of the metal due to the semi-metal conductivity nature of graphene, which would form galvanic circulate and favor the electrochemical oxidation of metals beneath graphene through an electron transport process [10,11,12]. Therefore, the modification of graphene and graphene-like materials based on its conductive nature is a promising substitution of graphene as a protective coating. For example, h-BN has been reported to be a good anti-corrosion coating for metals owing to its insulating characteristics [13,14,15]. In addition, heteroatoms doping of graphene, e.g., N-doped graphene (NG), can modify the electrical transport properties of graphene [16,17]. It was reported that the NG shows n-type semiconductor transport features, and results in a decline in electrical conductivity compared with pristine graphene (PG) [18]. Thus, doping graphene with N atoms is a potential route for improving the corrosion resistance of graphene as an effective barrier coating for metals [19].

As for the synthesis of NG, chemical vapor deposition (CVD) has been the most widely used method for the preparation of NG via the incorporation of N atoms into the structure of the graphene lattice during the formation of graphene in a nucleation-growth process. Normally, CH_4_ and NH_3_ are used as the carbon and nitrogen sources, respectively, for the CVD synthesis of NG. In addition, much attention has been focused on NG synthesis by mixing CH_4_/NH_3_ precursors through the CVD technique [18]. However, it is still of fundamental interest to exploit and develop new routes for the diversity synthesis of NG and investigate its anti-corrosion properties as a protective coating. Here, we developed a modified route for the synthesis of NG on a Cu substrate in two steps by the pre-implantation of N ions in a Cu substrate and the subsequent CVD growth of graphene. The pre-implanted N ions can dope CVD graphene through a series of processes from segregation to diffusion and finally to the incorporation of N into the graphene lattice by the substitution of C atoms. Moreover, by controlling the duration (dose) of the N ions implantation process, NG films with different N doping concentrations, which is an important factor to affect the corrosion resistance of NG films, could be obtained on the Cu surface. Based on this, the NG-coated Cu with a high N dose showed a better corrosion resistance than NG with a low N dose and PG-coated Cu for a long-term exposure to air at room temperature. These results demonstrate that the natural oxidation resistance of Cu coated by CVD-grown NG is highly dependent on their doped N concentration, providing insights into the corrosion mechanisms and correlations between graphene coatings and graphene-like materials with modified electrical properties.

## 2. Experimental Section

### 2.1. Synthesis of N-Doped Graphene (NG) and Pristine Graphene (PG)

NG films were grown on polycrystalline Cu foils with two steps: (Ⅰ) pre-implantation of N ions in Cu substrates and (Ⅱ) CVD growth of graphene on Cu pre-implanted with N ions. Cu foils were electrochemically polished and cleaned for N ions implantation. Different durations (dose) of processes (corresponding to PG, NG1, and NG2) were applied for the N ions implantation (PIII and D-700, TONGCHUANG, China). The duration of the process and detailed parameters are shown in Table 1. For the deposition of PG and NG films, all Cu foils were placed at the center of the heating furnace of the CVD system in a quartz tube. Cu(OAc)_2_ was used as a C source for clean graphene growth [20] and placed ~30 cm away from the heating center to ensure its decomposition at ~240 °C and the injection to the heating zone. The tube furnace was heated to 1050 °C with 150 sccm of Ar and 10 sccm of H_2_ in 20 min and was kept at this temperature for 5–20 min to grow PG or NG films.

### 2.2. Oxidation Test of PG- and NG-Coated Cu System

The PG- and NG-coated Cu samples were kept in an ambient environment for 0–180 days to study their long-term oxidation behaviors.

### 2.3. Characterization of PG- and NG-Coated Cu

PG and NG were confirmed through scanning electron microscopy (SEM, JEOL JSM-6390 A at 15 kV, Japan), Raman spectroscopy (HORIBA, using laser excitation in 532 nm, Japan), and X-ray photoelectron spectroscopy (XPS, AXIS ULTRADLD, Sweden). The corrosion degree of Cu was characterized via an optical microscope (OM, Carl Zeiss Microscopy Gmbh, Germany) and XPS.

## 3. Results and Discussion

**Figure 1:**
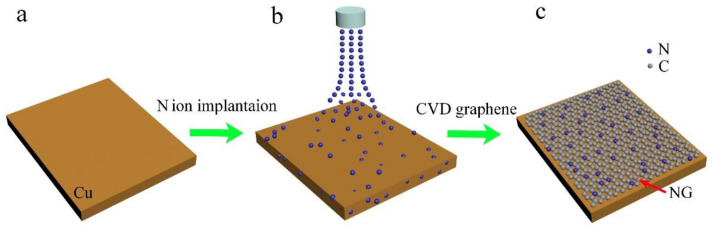
Diagram of N-doped graphene synthesis process: (**a**) polycrystalline Cu foils after cleaning; (**b**) pre-implantation of N ions in Cu substrates; (**c**) NG deposition on Cu surface.

**Figure 2:**
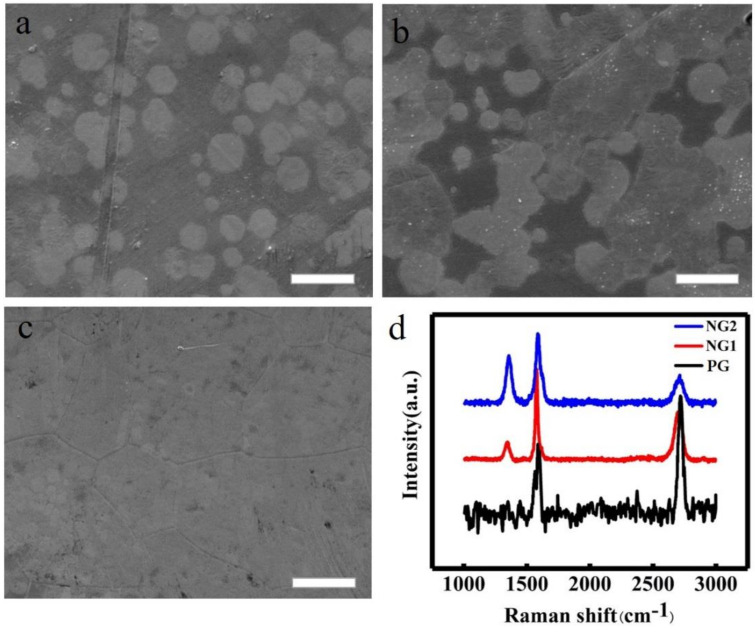
(**a**–**c**) SEM of fresh-prepared NG1-coated Cu in growth times of (**a**) 5, (**b**) 10, and (**c**) 20 min, respectively. Scale bar is 10 μm. (**d**) Raman spectra of fresh-prepared PG-, NG1-, and NG2-coated Cu.

The whole preparation processes of NG films are schematically shown in Figure 1. As shown, the pre-implanted N ions in the Cu substrate were used as the nitrogen source for the subsequent CVD formation of NG. It was supposed to go through a series of processes for N ions doping, including segregation, diffusion, and incorporation of N into the graphene lattice by the substitution of C during such a high-temperature CVD process. As the concentration of N ions implanted in the Cu foil should be a Gaussian distribution from the surface to a certain depth, the implanted N ions partly segregated and diffused to the surface, and then combined with C atoms decomposed from Cu(OAc)_2_, forming N-doped graphene with the assistance of a Cu catalyst. Thus, the manipulation of the duration (dose) of N pre-implantation could control the concentration of doped N in the formed NG. Apparently, the absence of N ions pre-implantation in the Cu substrate would produce PG films. Figure 2a–c show SEM images of fresh-prepared NG on Cu foil for different growth times based on the duration of 5 min of N ions pre-implantation, which also corresponds to NG1 (see Table 1). With the increase in the CVD growth time from 5 (Figure 2a), to 10 (Figure 2b), and finally to 20 min (Figure 2c), these randomly distributed NG nuclei islands grew together and eventually formed a polycrystalline NG film (Figure 2c). Raman spectroscopy was then employed to characterize NG1, NG2, and PG as an effective tool to detect the doping effects of graphene. As shown in Figure 2d, all Raman spectra showed G and 2D resonances at ~1583 and ~2710 cm^−1^, respectively. It is noted that there was no D peak in PG, which reflects the information of structural defects and partially disordered structures of the sp^2^ domains [21], indicating the high quality of the PG films. On the other hand, due to the doping of N atoms into graphene, the D-band of NG1 and NG2 began to appear at ~1356 cm^−1^. With the higher nitrogen content (NG2), the D-peak was higher, and a shoulder peak appeared on the right side of the G band at ~1630 cm^−1^ (D’-band), which is the Raman characteristic of crystal size or lattice distortion [22]. In other words, as the N concentration increased in NG, the intensities of the D and D’ bands increased and the intensity of the 2D peak decreased with respect to that of the G peak, which is ascribed to various bonding structures and defects after the introduction of N atoms [23,24].

**Figure 3:**
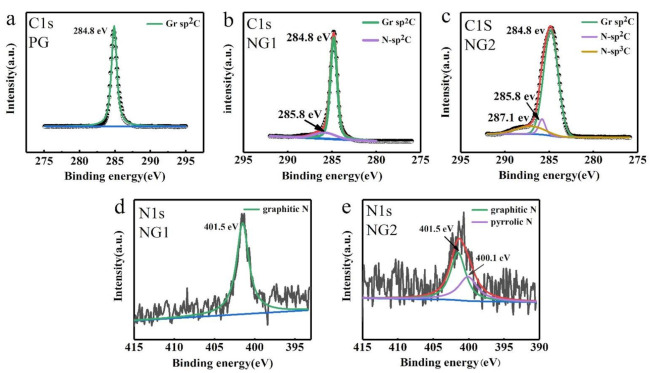
(**a**) XPS core-level C 1s spectra of fresh-prepared PG, (**b**) NG1, and (**c**) NG2; (**d**) XPS core-level N1s spectra of fresh-prepared NG1 and (**e**) NG2.

Further, chemical compositions of the PG-, NG1-, and NG2-coated samples were analyzed via X-ray photoelectron spectroscopy (XPS). The main peaks of the C 1s spectra of PG, NG1, and NG2 were all at 284.8 eV (see Figure 3a–c), which corresponds to the graphite-like sp^2^ hybridized carbon. In the PG sample, only this sp^2^ graphite peak can be discriminated (Figure 3a), which is reasonable for pristine graphene. In the NG1-coated Cu sample, besides the main graphite peak, a small peak located at 285.8 eV can be seen. However, for NG2 with higher nitrogen content, two tiny peaks at 285.8 and 287.1 eV can be clearly observed. These two peaks represent two C-N bond structures of sp^2^ and sp^3^ hybrid carbon caused by N atom substitution [25,26,27], respectively. The N 1s spectra of the NG1-coated Cu samples (Figure 3d) show that the nitrogen atoms were composed of graphite-N (401.5 eV). That means that the N atoms completely replaced the C atoms in the conjugated honeycomb lattice [18], while NG2 with the higher nitrogen content demonstrated a broader N 1s peak with graphitic-N (401.5 eV) and pyrrolic-N (400.1 eV) [28,29,30,31] (Figure 3e) due to the substitution of the N atom at the defects and/or the edges of the graphene sheets [18]. The higher graphitic-N proportion illustrates that the pre-implanted N ions inside Cu were mainly incorporated into the graphene layer by the substitution doping of C atoms during the CVD growth. In addition, the N/C atomic ratio of NG1-coated Cu and NG2-coated Cu was estimated to be ~2.44% and 6.32%, respectively, according to the XPS results [32].

**Figure 4:**
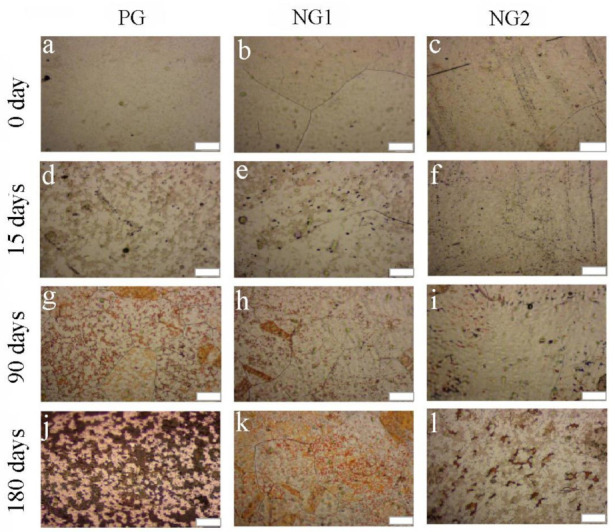
OM of the samples after exposure in air. (**a**–**c**) OM of PG-, NG1-, and NG2-covered Cu freshly prepared, respectively; (**d**–**f**) OM of PG-, NG1-, and NG2-coated Cu left in the ambient for 15 days, respectively; (**g**–**i**) OM of PG-, NG1-, and NG2-coated Cu left in the ambient for 90 days, respectively; (**j**–**l**) OM of PG-, NG1-, and NG2-coated Cu left in the ambient for 180 days, respectively. Scalebar is 20 μm. These photos were taken under uniform exposure conditions.

In order to investigate the corrosion resistance of synthesized NG, long-term ambient oxidation was performed. For comparison, the copper foils coated with PG, NG1, and NG2 were placed in glass plates at room temperature (about 20 °C) for up to 180 days. The humidity of the ambient was unstable but low. Figure 4 shows the optical micrographs (OM) of PG-, NG1-, and NG2-coated Cu foils after 0 day (freshly prepared), 15 days, 90 days, and 180 days of exposure in air, respectively. As shown in Figure 4a–c, all the fresh-prepared samples showed a similar surface to that of bare Cu. After 15 days, irregular and uneven small pieces of Cu oxides began to appear on the PG-coated Cu and NG1-coated Cu surfaces (Figure 4d,e). However, NG2-coated Cu still maintained a relatively good metallic surface (Figure 4f). After 90 days, the PG-coated Cu was oxidized with an oxidation area up to ~50% (Figure 4g). Meanwhile, the oxidation of NG1-coated Cu was more serious than that of NG2-coated Cu (Figure 4 h,i). After 180 days, entire regions of the PG-coated Cu appeared oxidized, and parts of the black area were heavily oxidized. Obviously, the galvanic corrosion between PG and Cu formed and accelerated the corrosion of copper during such a long-term ambient oxidation. In comparison, much lesser Cu oxidation took place on the NG1- and NG2-coated Cu surface. Moreover, with the increase in doped N concentration from NG1 to NG2, NG2 demonstrated a better anti-corrosion protection for Cu (see Figure 4k,l).

**Figure 5:**
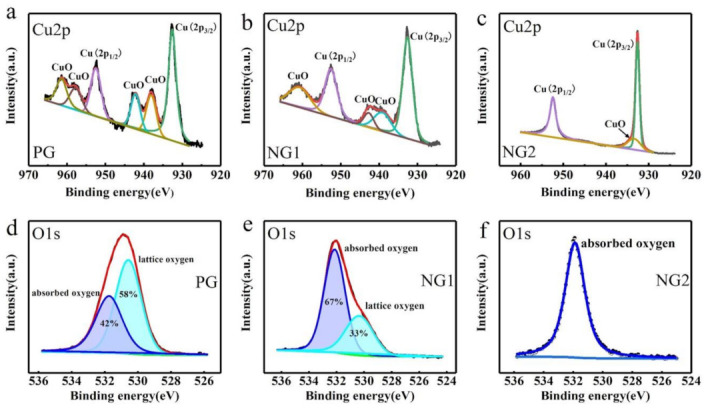
(**a**) XPS core-level Cu 2*p* spectra of PG-, (**b**) NG1-, and (**c**) NG2-coated Cu after 90 days of exposure. (**d**) XPS core-level O 1s spectra of PG-, (**e**) NG1-, and (**f**) NG2-coated Cu after 90 days of exposure.

To further determine the degree of oxidation of Cu, we performed XPS tests on the PG-, NG1-, and NG2-coated Cu exposed to air for 90 days. Characteristic peaks of Cu 2p_2/3_ and Cu 2p_1/2_ at binding energies of 932.6 and 952.5 eV can be observed in the three spectra (Figure 5a–c), respectively. It can be clearly seen from Figure 5a–c that the peak widths of these two peaks decreased, which indicates that the oxidation degree of Cu also decreased. The existence of CuO can be proved by two small satellite peaks next to each main peak, respectively, at binding energies of 961.5, 957.9, 942.6, and 938.1 eV [11,12] (Figure 5a). The high intensity of the four small satellite peaks proves the serious corrosion of PG-coated Cu. In Figure 5b, there are three satellite peaks with relatively low intensities at 961.1, 942.8, and 939.6 binding energies, proving that there was relatively less CuO in NG1-coated Cu. In Figure 5c, there is only a tiny satellite peak at a binding energy of 933.5 eV, proving that CuO was extremely rare in NG2-coated Cu. Characteristic peaks of absorbed oxygen (O^2−^ ion or O^−^ ion formed by the adsorption of oxygen molecules in the gas phase on the surface of metal oxides by chemisorption) at a binding energy of 530.6 eV [19,33] can be observed in the three patterns (Figure 5d–f). We know that the ratio of the peak area reflects the ratio of the bonding structure. The area of oxygen in the lattice (bonded to the metal) reached up to 58% in Figure 5a, indicating that PG-covered Cu was heavily oxidized. With the addition of N atoms, the area of lattice oxygen began to decrease from 33% (NG1) to nearly 0% (NG2), as seen in Figure 5e,f, which means that the degree of oxidation of Cu decreased. We cannot see the peak of lattice oxygen in NG2-coated Cu (Figure 5f), probably because the lattice oxygen is too weak to cause a change in the binding energy of the inner shell, and the spectral lines do not shift. These data indicate that after 90 days of exposure, PG-covered Cu severely oxidized, NG1-covered Cu also partially oxidized, and NG2-covered Cu only slightly oxidized to a low degree.

**Figure 6:**
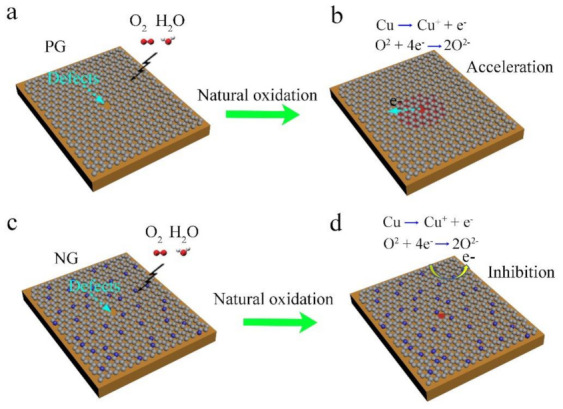
(**a**,**b**) Diagram of galvanic corrosion mechanism of copper by PG in long-term; (**c**,**d**) diagram of protection mechanism of copper by N-doped graphene in long-term.

These experimental results and data analysis intuitively show that the graphene doped with N improved the anti-corrosion performance of graphene, and with the increase in N concentration, the anti-corrosion performance of NG for Cu improved. As we know, graphene is a good protective coating for Cu when it is applied to severe oxidation (e.g., heating oxidation) owing to its impermeability to most of the corrosive species. However, over a long period of time, O_2_ and H_2_O corrosive species can come into contact with Cu through various graphene defects [34,35,36] and form galvanic corrosion between graphene and the Cu substrate underneath due to the electron transport via the conductive graphene layer (see Figure 6a,b). However, the incorporation of N atoms into the graphene here could decrease the conductivity of the graphene. In addition, the charge carrier transfer between NG and Cu was then inhibited, reducing the electrochemical corrosion rate (Figure 6c,d). As a result, with the increase in N atom concentration in NG, the corrosion rate of Cu was slower and the protection effect improved.

## 4. Conclusions

In conclusion, a new method for the synthesis of N-doped graphene was developed here by pretreating Cu with an N ions implantation method and the subsequent growth of N-doped graphene on pretreated Cu by CVD. Then, the long-term protective effect of N-doped graphene with different doping concentrations for Cu corrosion was tested by ambient oxidation. The results showed that NG-coated Cu could improve its corrosive barrier with the increase in doped N concentration in NG, and it demonstrated much better protective properties for Cu in the long term than those of PG-coated Cu, which could be ascribed to the reduction in conductivity of NG and inhibition of the electrochemical reaction between the NG coating and Cu substrate in the long term. These findings could provide insights into corrosion mechanisms of the graphene coating and correlate its conductive nature based on heteroatoms doping, which is a potential route for improving the corrosion resistance of graphene as an effective barrier coating for metals.

## Figures and Tables

**Table 1:** Process parameters of N ions implantation.

Item	PG	NG1	NG2
N_2_ flow (sccm)	NA ^1^	25	25
Pressure (Pa)	NA	5.1 × 10^−2^	5.1 × 10^−2^
Bias voltage (kV)	NA	−15.3	−15.3
Radiofrequency power (W)	NA	300	300
Duration (min)	NA	5	15

^1^ NA = not applicable.

## Data Availability

Data are contained within the article.
